# ﻿Two new species of the genus *Laena* (Coleoptera, Tenebrionidae, Lagriinae) from northern Sichuan in China based on morphological and molecular data

**DOI:** 10.3897/zookeys.1173.103125

**Published:** 2023-08-01

**Authors:** Zhonghua Wei, Guodong Ren

**Affiliations:** 1 The Key Laboratory of Southwest China Wildlife Resources Conservation of the Ministry of Education, College of Life Sciences, China West Normal University, 637009, Nanchong, Sichuan Province, China China West Normal University Nanchong China; 2 The Key Laboratory of Zoological Systematics and Application of Hebei Province, College of Life Sciences, Hebei University, 071002, Baoding, Hebei province, China Hebei University Baoding China

**Keywords:** Darkling beetles, *Laena*, Laenini, molecular species delimitation, new taxa, taxonomy

## Abstract

In this study, the *Laena* species from northern Sichuan Province were reviewed using a combination of molecular and morphological datasets. Three molecular methods for species delimitation were firstly used in the genus *Laena* to explore species boundaries. The results show that the number of morphospecies and putative species using Automatic Barcode Gap Discovery (ABGD) is consistent. Based on the results, two new species are described and illustrated: *Laenamounigouica***sp. nov.** and *Laenadentithoraxa***sp. nov.** New materials and distribution of 10 *Laena* species are also provided in this study. This work also provides valuable molecular data for species identification and phylogenetic analyses of the genus *Laena* and subfamily Lagriinae.

## ﻿Introduction

The tribe Laenini Seidlitz, 1896 includes 13 genera distributed in Palaearctic, Oriental, Afrotropical, and Neotropical regions (Bouchard et al. 2021). There are two genera, *Laena* Dejean, 1821 and *Hypolaenopsis* Masumoto, 2001, recorded from China. The genus *Hypolaenopsis*, including five species, is an endemic genus distributed west of the Qingling Mountains (Schawaller 2001, 2008). To date, the genus *Laena* includes more than 350 species distributed in the Palaearctic, Oriental, and Afrotropical regions, and 126 species have been recorded from China (Schawaller 2021; Wei et al. 2021). Over the past hundred years, some tenebrionid taxonomists have made significant contributions to the development of the genus *Laena* in China, especially Schawaller and his colleagues (Schawaller 2001, 2008, 2021; Schawaller and Aston 2017; Schawaller and Bellersheim 2023), who have described more than 80 species from China. During the past decade, members of Guodong Ren’s Laboratory have also been devoted to the taxonomy of the genus *Laena* from China (Zhao and Ren 2011, 2012a, b; Wei and Ren 2019a, b; Wei et al. 2020, 2021). *Laena* species are flightless and well adapted to humid environments.

In China, the Hengduan Mountains region has the highest *Laena* species richness based on the previous reports (Schawaller 2001, 2008; Zhao and Ren 2012a, b; Wei et al. 2020), followed by the Qinling Mountains region. Sichuan Province is located between these two regions and includes many mountains, implying that it may also contain abundant *Laena* species. To explore the detailed distribution of *Laena* species in Sichuan Province, one of the authors collected tenebrionid specimens in northern Sichuan Province in July and August 2022. During the identification of *Laena* specimens from this investigation, two undescribed species were discovered. In this study, these two new species are described and illustrated, and the *COI* of new species and known species are also provided for molecular delimitation.

## ﻿Materials and methods

### ﻿Morphological examination

In this study, all the specimens were collected from northern Sichuan Province of China in July and August 2022. They are preserved in 95% alcohol at −24 °C for further use and deposited in the specimen collection of China West Normal University, Nanchong, China (**CWNU**). These specimens were examined using an Olympus SZX10 microscope. The figures were taken with a Canon EOS 9D Mark III camera connected to a Laowa FF 25 mm F2.8 Ultra Macro 2.5–5× lens and edited in Photoshop CC 2019. Although a key to separate *Laena* species from Sichuan Province was provided by Wei et al. (2020), an updated key is not provided in this study because several undescribed species with single specimen are known from the same region and some species are described by Schawaller (2021).

### ﻿Taxon sampling, DNA extraction, PCR amplification, and sequencing

All the specimens were collected by sifting the litter of forests in summer. Whole genomic DNA of 25 *Laena* and three *Hypolaenopsis* individuals was extracted from leg and thorax muscle tissues using the Ezup Column Animal Genomic DNA Purification Kit (Shanghai, China) following the manufacturer’s instructions. All polymerase chain reactions (PCR) were conducted using Trident 960 thermal cycler (Heal Force, Shanghai, China) under the following conditions: initial denaturation for 4 min at 94 °C, 35 cycles of 1 min at 94 °C, 1 min at 72 °C, and a final extension at 72 °C for 8 min. The bidirectional sequencing was conducted by Sangon Biotech Co. Ltd (Shanghai, China). The detailed information of all samples is provided in Suppl. material 1. The species *Anaedusbrunneus* was used as the outgroup in this study.

### ﻿Phylogenetic analyses

The newly generated sequences were checked and corrected using SeqMan v. 7.1.0 and were edited using BioEdit v. 7.1.11 (Hall 1999). All the sequences including outgroup were aligned using Clustal W (Thompson et al. 1994) and then the aligned sequences were trimmed using trimmAl v. 1.2 (Capella-Gutiérrez et al. 2009). The test of substitution saturation for *COI* gene was performed with DAMBE (Xia 2017). ModelFinder (Kalyaanamoorthy et al. 2017) was used to calculate the best substitution models in the maximum-likelihood (ML) analyses. For the maximum-likelihood analyses, IQ-TREE v. 1.6.6 (Guindon et al. 2010) was used to construct the phylogenetic tree.

To explore the boundaries of *Laena* species, three methods were used to determine the molecular species delineation. The online tools Automatic Barcode Gap Discovery (ABGD) and Assemble Species by Automatic Partitioning (ASAP) (Puillandre et al. 2021) were carried out on the *COI* gene. Another online tool, Poisson Tree Processes (PTP) (Zhang et al. 2013), was also used to analyze the species delimitation based on the ML tree. During processing with this approach, the default settings were used, and the outgroups were removed.

## ﻿Results

### ﻿Phylogenetic analysis and species delimitation

**Figure 1.**
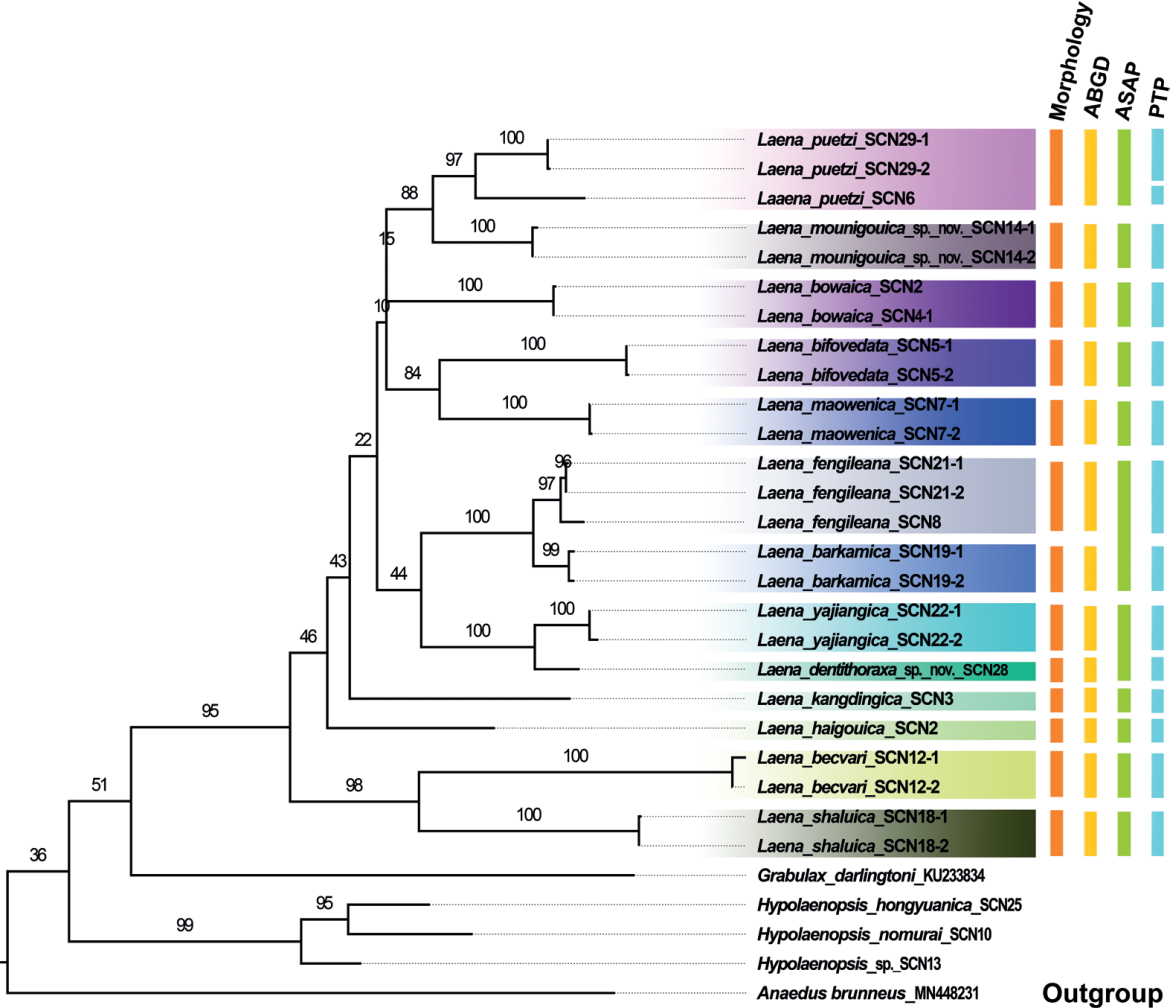
Maximum-likelihood (ML) tree based on *COI* sequences within the tribe Laenini. Support for each node is represented by ultrafast bootstrap values. The results of ABGD, ASAP, and PTP are also presented in ML tree.

In this study, 28 new sequences of 13 *Laena* species and three *Hypolaenopsis* species from northern Sichuan were generated. The best-fit model is GTR+F+I+G4 chosen according to Bayesian Information Criterion. The ML tree suggested that all morphological species are monophyletic, with different bootstrap values varied from 97 to 100 (Fig. 1). All Laenini species converged together as an independent clade with low-value support (ML = 36). In the present study, the genus *Laena* was close to the genus *Grabulax* in the tribe Laenini. *Laenamounigouica* sp. nov. clustered together with *L.puetzi*, while *L.dentithoraxa* sp. nov. clustered together with *L.yajiangica*. Thirteen molecular operational taxonomic units (**MOTUs**) were estimated by ABGD for 28 samples (Fig. 1), and 13 morphological species were well supported by the ABGD result; morphological assumptions and molecular results of ASAP and PTP differ only for *L.fengileana*, *L.barkamica*, and *L.puetzi*.

### ﻿Taxonomy

**Tribe Laenini Seidlitz, 1895**

**Genus *Laena* Dejean, 1821**

#### ﻿ Laena mounigouica sp. nov.

Taxon classificationAnimaliaColeopteraTenebrionidae

B6A54687-C0C6-5413-9BBF-2F0FED193861

https://zoobank.org/119947B4-245C-4710-8873-22D97E

Fig. 2

##### Type locality.

China, Sichuan Province, Songpan County, Mounigou.

##### Type specimens.

***Holotype***: ♂, China, Sichuan, Songpan County, Mounigou, Tuguanzhai, elev. 2978 m, 2022.VII.21, Zhonghua Wei leg., CWNU. ***Paratype***: 2♂2♀, the same data of holotype, CWNU.

##### Other examined materials.

1♂1♀ (in ethanol), China, Sichuan, Songpan County, Mounigou, Tuguanzhai, elev. 2978 m, 2022.VII.21, Zhonghua Wei leg., CWNU.

##### Diagnosis.

This is the second species from Mounigou in Songpan County. *Laenalangmusica* Schawaller, 2001 was recorded from Zhaga Waterfall in Mounigou by Schawaller (2008), but the new species can be easily separated by the following characteristics: (1) body small; (2) pronotum with dense punctures; (3) elytral intervals with dense irregular micro-punctures; (4) all tibiae hooked in males; (5) aedeagus with constricted apicale. This new species also similar to *L.hengduanica* based on the key provided by Wei et al. (2020), but it can be distinguished by the following characters: (1) smaller body (length 4.0–4.3 mm); (2) elytral intervals with dense irregular micro-punctures; (3) male tibiae without granules.

##### Description.

**Figure 2.**
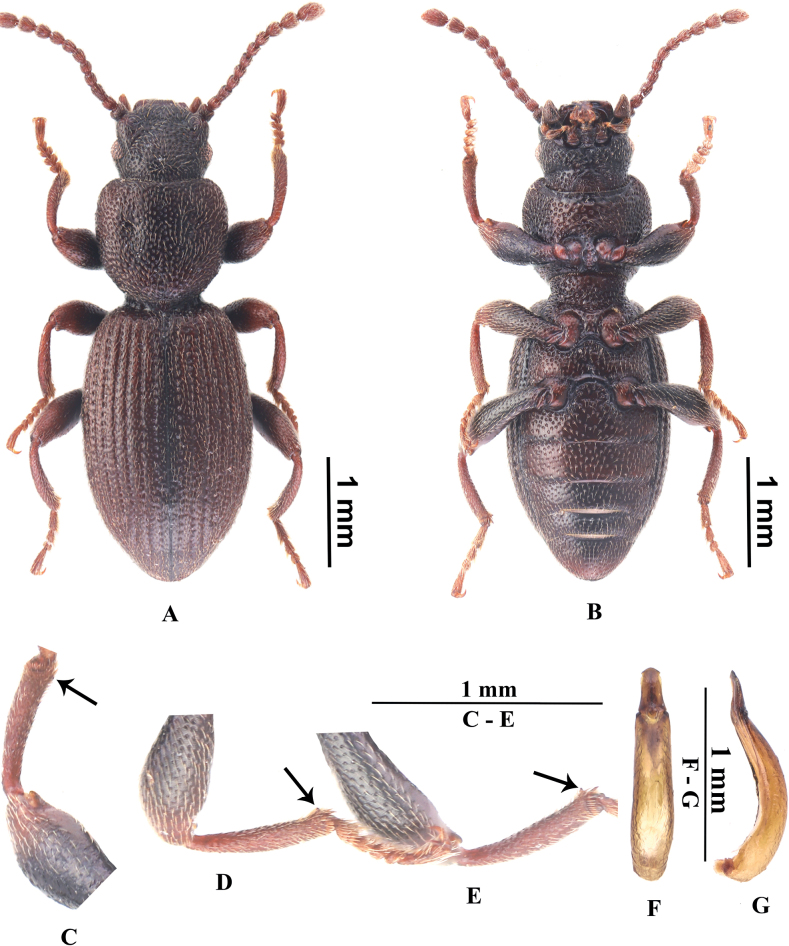
*Laenamounigouica* sp. nov., holotype **A, B** dorsal and ventral views **C** profemur and protibia, in ventral view **D** mesofemur and mesotibia, in ventral view **E** metafemur and metatibia, in ventral view **F, G** aedeagus, in dorsal and lateral views.

**Male. *Body*** length 4.0–4.3 mm, width 1.4–1.6 mm. Body (Fig. 2A, B) blackish brown; dorsal surface shining, with dense punctures bearing short setae.

***Head*** trapezoidal, cranial punctate contradicts smooth, with dense, large punctation bearing dense, short setae. Genae distinctly raised, surface without punctures in apical part. Eyes ovate, not reduced, prominent. Epistome nearly trapezoidal, anterior margin weakly emarginated; surface slightly convex, with shallow, large punctures, each lateral part with three longer setae. Fronto-clypeal suture distinct, not depressed, straight at middle. Frons even. Vertex weakly convex at middle, posterior-lateral part of surface each with one longer seta. Antennae moderate, reaching basal margin of pronotum when directed backwards, antennomeres I distinct thicker than II–VIII, antennomere III approximately 1.5 times as long as antennomere II, relative ratio of the length of antennomeres II–XI as follows: 0.65:0.98:0.73:0.64:0.72:0.72:0.78:0.90:1.03:1.68.

***Pronotum*** (Fig. 2A) widest at anterior 1/3, approximately 1.1 times as wide as long and 1.3 times as wide as head; anterior margin distinctly emarginate at middle, not beaded; lateral margins widened anteriorly and strongly convergent from anterior 1/3 to anterior margin, beading not distinct; basal margin not beaded, not bent downwards; disc evenly convex, with a pair of shallow pits at middle and a longitudinal shallow groove along with middle line, surface with dense punctures bearing short setae, distance between punctures 0–1 times puncture diameter; anterior angles rounded, slightly produced; posterior angles rounded, not produced. Prothoracic hypomera with similar punctures and setae. Prosternal process widest at apices, bent downwards behind coxae; disc impressed between coxae, with dense, large punctures bearing shorter setae.

***Elytra*** elongate-oval, widest at middle, approximately 1.6 times as long as wide; lateral sides nearly paralleled in middle; humeral angles rounded. Elytral surface shiny, with rows of punctures without striate; punctures in rows as large as those of pronotum; intervals weakly smooth and glabrous, with very small punctures nearly invisible, interval IX with three setigerous pores bearing longer setae. Elytral apices prolonged in dorsal view, apex obtuse.

***Abdomen*** long ovoid, widest in middle, 1.8 times as long as wide. Surface convex, glabrous, with smaller punctures than those of dorsal surface; posterior part of sternite IV distinctly convex transversely before posterior margin.

***Legs*** slender, surface shining, with smaller punctures bearing short setae. All femora (Fig. 2C–E) without teeth near apex on inner sides. All tibiae slightly curved and hooked (Fig. 2C–E) at inner apex.

***Aedeagus*** (Fig. 2F, G) subfusiform, length 1.2–1.3 mm, width 0.2–0.3 mm. Parameres with apex blunt, widest at base, lateral margins evenly convergent towards apices, significantly constricted apex at lateral sides of apices.

##### Sexual dimorphism.

All tibiae of female not hooked at inner apex.

##### Distribution.

China: Sichuan.

##### Etymology.

The name of this species is based on type locality.

##### Biology.

The *Laenamounigouica* sp. nov. was collected in leaf litter of coniferous forests and mixed coniferous and broad-leaved forests from Mounigou, which is the second species found at elevation between 2900–3120 m in this scenic spot.

#### ﻿ Laena dentithoraxa sp. nov.

Taxon classificationAnimaliaColeopteraTenebrionidae

45455285-BEAE-541B-B116-CC03BB770491

https://zoobank.org/65011905-3C2F-4010-9D56-122F6816E952

Fig. 3

##### Type locality.

China, Sichuan Province, Yajiang County, Yizhan.

##### Type specimens.

***Holotype***: ♂, China, Sichuan, Yajiang Yizhan, elev. 2800 m, 2022.VIII.6, Zhonghua Wei leg., CWNU. ***Paratype***: 2♂2♀, China, Sichuan, Yajiang Yizhan, elev. 2800 m, 2022.VIII.6, Zhonghua Wei leg., CWNU.

##### Other examined materials.

1♀, in ethanol, China, Sichuan, Yajiang Yizhan, elev. 2800 m, 2022.VIII.6, Zhonghua Wei leg., CWNU.

##### Diagnosis.

This new species should belong to the *L.yajiangica* species-group: *L.yajiangica* Schawaller, 2001, *L.yulongica* Schawaller, 2001, *L.bowaica* Schawaller, 2001, and *L.schuelkei* Schawaller, 2001. This new species is similar to *L.yajiangica* Schawaller, 2001 (Fig. 4A, B) in body shape and crenulated lateral margins of the pronotum, but it can be easily distinguished by all femora having distinct teeth near the inner apex and an aedeagus with constricted apices.

##### Descriptions.

**Figure 3.**
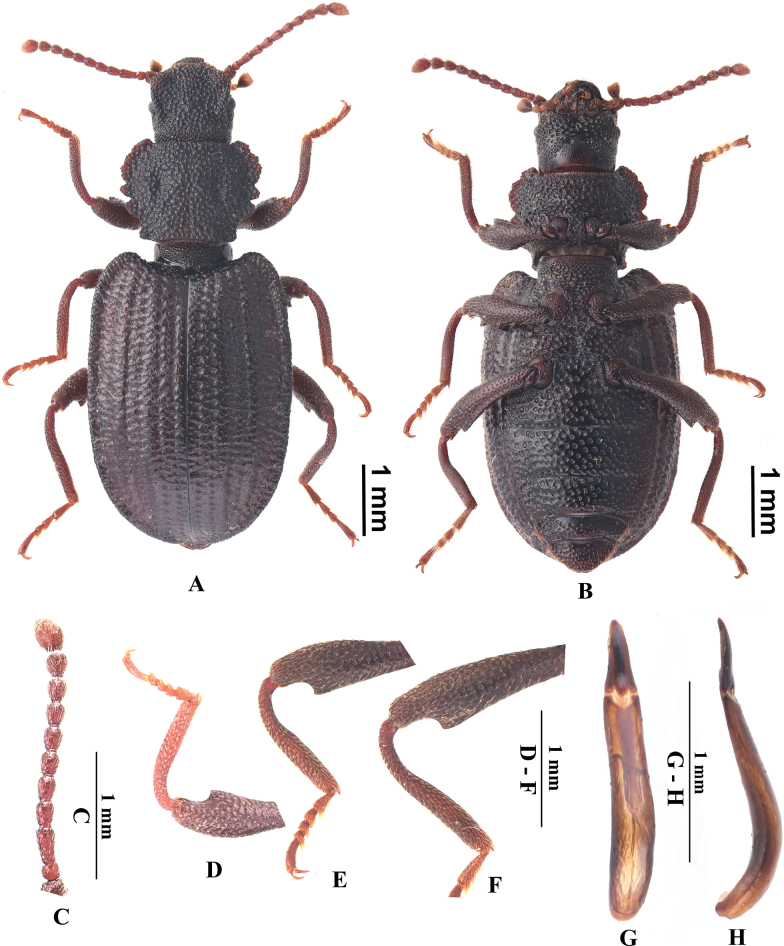
*Laenadentithoraxa* sp. nov., paratype, male **A, B** dorsal and ventral views **C** antenna **D** profemur and protibia, in ventral view **E** mesofemur and mesotibia, in ventral view **F** metafemur and metatibia, in ventral view **G, H** aedeagus, in dorsal and lateral views.

**Male. *Body*** length 8.9–9.1 mm, width 3.0–3.2 mm. Body (Fig. 3A, B) blackish brown, surface coarse and dull, with dense and large punctures bearing very sparse, short setae; pronotal surface with irregular wrinkles.

***Head*** trapezoidal, cranial surface coarse, with dense, large punctation bearing very sparse, short setae. Genae strongly raised, surface with dense and large punctures including apical part. Eyes ovate, not reduced and prominent. Epistome nearly trapezoidal, anterior margin slightly emarginated, surface with dense, large punctures. Fronto-clypeal suture distinctly depressed. Frons convex at middle, surface with deep longitudinal groove at lateral side. Antennae (Fig. 3C) short, reaching basal 1/3 of pronotum when directed backwards, antennomeres I distinct thicker than II–IX, antennomere III approximately 2.1 times as long as antennomere II, relative ratio of the length of antennomeres II–XI as follows: 0.64:1.37:0.86:0.86:0.92:0.89:0.89:0.92:1.01:1.60.

***Pronotum*** (Fig. 3A) widest at anterior 1/3, approximately 1.3 times as wide as long and 1.3 times as wide as head. Anterior margin evenly emarginate; lateral margins strongly crenulated, not beaded; basal margin nearly straight, not bent downwards; disc convex, descendent laterally, surface with dense and large fused punctures, bearing sparse and short setae, interspace between punctures strongly raised, with a pair of deep pits at middle and a shallow groove along with middle line. Anterior angles acute, strongly produced; posterior angles near rectangular, not produced. Prothoracic hypomera with punctures as large as those on pronotal disc. Prosternal process widest at apices, bent downwards behind coxae; surface with dense, large punctures bearing sparse, short setae. Meso- and metaventrite with dense, large punctures bearing short setae.

***Elytra*** (Fig. 3A) elongate-oval, widest at middle, approximately 1.4 times as long as wide; lateral sides nearly paralleled from base to apical 1/4; humeral angles acute, strongly produced. Elytral surface roughened, flat, with sparse, short setae, with rows of punctures without striae; punctures in rows as large as those on pronotum, those on intervals very small nearly invisible; intervals III and V slightly convex, interval VII strongly convex, intervals VIII and IX invisible in dorsal view, interval IX with two setigerous pores bearing longer setae. Elytral apices prolonged in dorsal view, apex obtuse.

***Abdomen*** long ovoid, 1.8 times as long as wide, widest in middle. Surface convex, with dense and large punctures; posterior part of sternite IV strongly convex along with posterior margin; punctures on sternite IV and V smaller than those of sternite I–III.

***Legs*** slender, surface coarse, with smaller punctures bearing moderate setae. All femora (Fig. 3D–F) with distinct obtuse teeth near apex on inner sides. Base of protibiae more curve than that of meso- and metatibiae; all tibiae not hooked at inner apex.

***Aedeagus*** (Fig. 3G–H) length 1.7–1.8 mm, width 0.2–0.3 mm. Parameres elgate trapezoid, with constricted apex widest at base, lateral margins evenly convergent towards apices in dorsal view.

##### Sexual dimorphism.

These female specimens without significant differences.

##### Distribution.

China: Sichuan.

##### Etymology.

The name of this species is based on the pronotum with well-developed teeth on lateral margins.

#### ﻿ Laena barkamica

Taxon classificationAnimaliaColeopteraTenebrionidae

Schawaller, 2008

533083EC-D273-59D3-A102-40D4535E486D

##### Examined materials.

2♂2♀, in ethanol, China, Sichuan, Heishui, Yangyong, Hade, elev. 2600 m, 2022.VII.26, Zhonghua Wei leg., CWNU.

##### Distribution.

China: Sichuan.

#### ﻿ Laena becvari

Taxon classificationAnimaliaColeopteraTenebrionidae

Schawaller, 2001

CCC405DB-0C80-5B0A-8DB0-E08DA08C1E4F

##### Examined materials.

3♀, China, Sichuan, Litang, Junba, elev. 3050 m, 2022.VIII.7, Zhonghua Wei leg., CWNU.

##### Distribution.

China: Sichuan.

#### ﻿ Laena bowaica

Taxon classificationAnimaliaColeopteraTenebrionidae

Schawaller, 2001

17E6A10B-9D31-5325-A7BF-E012B4A12A3F

Fig. 4C

##### Examined materials.

2♂2♀ (1♂1♀, in ethanol), China, Sichuan, Danba, Bianerxiang, Erwacao Village, elev. 2470 m, 2022.VIII.1, Zhonghua Wei leg., CWNU; 1♀, in ethanol, China, Sichuan, Danba, Dongguzhen, elev. 2360 m, 2022.VIII.2, Zhonghua Wei leg., CWNU; 1♂, in ethanol, China, Sichuan, Danba, Yongxi Village, elev. 2200m, 2022.VIII.2, Zhonghua Wei leg., CWNU.

**Figure 4.**
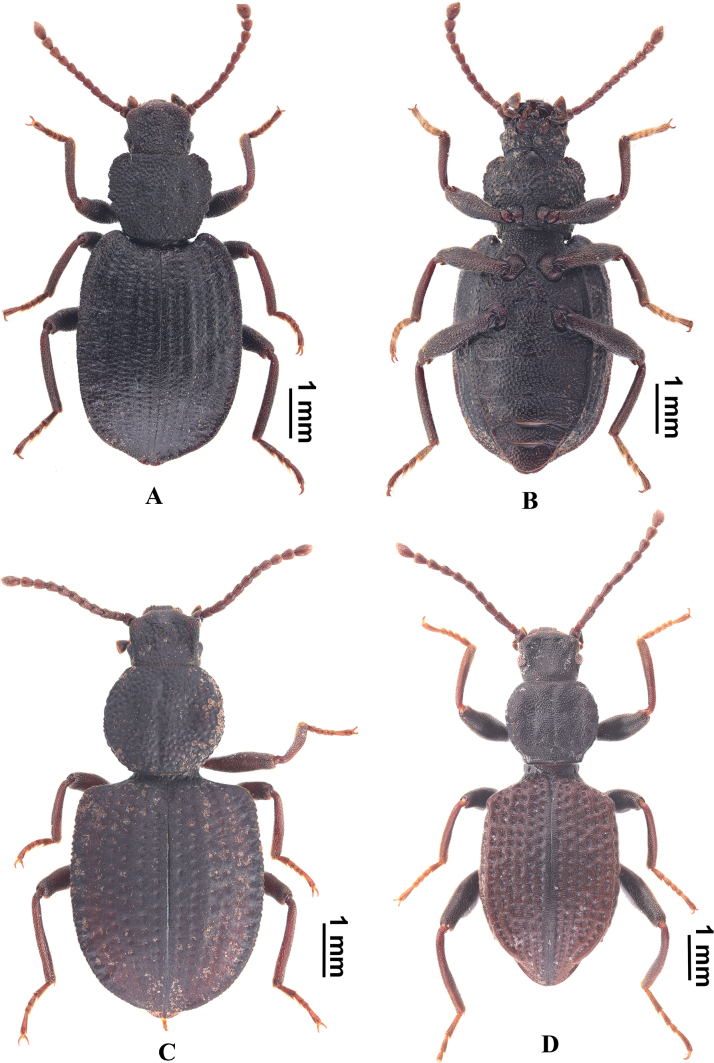
Illustration of *Laena* species **A, B***L.yajiangica* Schawaller, 2001, dorsal and ventral views **C***L.bowaica* Schawaller, 2001 **D***L.maowenica* Schawaller, 2008.

##### Distribution.

China: Sichuan.

#### ﻿ Laena fengileana

Taxon classificationAnimaliaColeopteraTenebrionidae

Masumoto, 1996

A970211B-E624-5949-B561-BD521ADB9527

##### Examined materials.

3♂2♀, China, Sichuan, Songpan, Mounigou, Shangzhai Village, elev. 3070 m, 2022.VII.22, Zhonghua Wei leg., CWNU; 4♂, China, Sichuan, Songpan, Chuanzhusi, Mayi Village, 2022.VII.25, Zhonghua Wei leg., CWNU; 2♂, in ethanol, China, Sichuan, Songpan, Huanglongxiang, Dawan Village, elev. 2920 m, 2022.VII.23, Zhonghua Wei leg., CWNU.

##### Distribution.

China: Shaanxi, Gansu, Sichuan.

#### ﻿ Laena haigouica

Taxon classificationAnimaliaColeopteraTenebrionidae

Schawaller, 2001

EBFE4409-0F18-5F7E-966B-FCCC2B29CBD0

##### Examined materials.

1♂, in ethanol, China, Sichuan, Songpan, Huanglongxiang, Dawancun, elev. 2920 m, 23.VII.2022, Zhonghua Wei leg., CWNU.

##### Distribution.

China: Sichuan.

#### ﻿ Laena kangdingica

Taxon classificationAnimaliaColeopteraTenebrionidae

Schawaller, 2001

D7626A91-6E4B-5A3C-85FC-7BFB50ABB538

##### Examined materials.

2♀ (1♀, in ethanol), China, Sichuan, Yajiang, Waduozhen, elev. 2600 m, 2022.VIII.5, Zhonghua Wei leg., CWNU.

##### Distribution.

China: Sichuan.

#### ﻿ Laena maowenica

Taxon classificationAnimaliaColeopteraTenebrionidae

Schawaller, 2008

08B65187-ECCF-5949-88F4-E4C69150AB3D

Fig. 4D

##### Examined materials.

4♂1♀ (2♂, in ethanol), China, Sichuan, 6 KM Eastern Mao County, elev. 1896 m, 2022.VII.20, Zhonghua Wei leg., CWNU.

##### Distribution.

China: Sichuan.

#### ﻿ Laena puetzi

Taxon classificationAnimaliaColeopteraTenebrionidae

Schawaller, 2001

C99FED26-C50D-58C9-9E3C-353243483390

##### Examined materials.

4♂2♀ (2♂, in ethanol), China, Sichuan, Barkman, Shaerzong, Dazatou Village, elev. 2690 m, 2022.VII.29, Zhonghua Wei leg., CWNU; 1♂, 1♀ (in ethanol), China, Sichuan, Barkman, Suomoxiang, Kanzhulin Village, 2022.VII.27, Zhonghua Wei leg., CWNU; 1♂, China, Sichuan, Yajiang, Waduozhen, elev. 2600 m, 2022.VIII.5, Zhonghua Wei leg., CWNU; 1♀ (in ethanol), China, Sichuan, Jinchuan, Dusongxiang, Dusonggou, elev. 2264 m, 2022.VII.31, Zhonghua Wei leg., CWNU.

##### Distribution.

China: Sichuan.

#### ﻿ Laena shaluica

Taxon classificationAnimaliaColeopteraTenebrionidae

Schawaller, 2001

920A4D4C-CBA3-51E4-AAA1-56AFF2B0FB98

##### Examined materials.

2♂1♀, in ethanol, China, Sichuan, Yajiang, Waduozhen, Ridui Village, elev. 3100 m, 2022.VIII.5, Zhonghua Wei leg., CWNU; 3 exs, China, Sichuan, Yajiang, Waduozhen, Ridui Village, elev. 3100 m, 2022.VIII.5, Zhonghua Wei leg., CWNU.

##### Distribution.

China: Sichuan.

#### ﻿ Laena yajiangica

Taxon classificationAnimaliaColeopteraTenebrionidae

Schawaller, 2001

52D70D01-F93F-5E08-871E-3DD54FC00441

Fig. 4A, B

##### Examined materials.

1♂2♀, China, Sichuan, Daofu, Xiatuoxiang, Yiwu Village, 2780 m, 2022.VIII.4, Zhonghua Wei leg., CWNU.

##### Distribution.

China: Sichuan.

## Supplementary Material

XML Treatment for Laena mounigouica

XML Treatment for Laena dentithoraxa

XML Treatment for Laena barkamica

XML Treatment for Laena becvari

XML Treatment for Laena bowaica

XML Treatment for Laena fengileana

XML Treatment for Laena haigouica

XML Treatment for Laena kangdingica

XML Treatment for Laena maowenica

XML Treatment for Laena puetzi

XML Treatment for Laena shaluica

XML Treatment for Laena yajiangica

## ﻿Additional information

### Author ORCIDs

Zhonghua Wei https://orcid.org/0000-0001-7349-9939

Guodong Ren https://orcid.org/0000-0001-5808-9122

### Data availability

All of the data that support the findings of this study are available in the main text or Supplementary Information.

## Supplementary materials

Supplementary material 1 Supplementary information Data type multimedia (ZIP file) https://binary.pensoft.net/file/886675This dataset is made available under the Open Database License (http://opendatacommons.org/licenses/odbl/1.0/). The Open Database License (ODbL) is a license agreement intended to allow users to freely share, modify, and use this Dataset while maintaining this same freedom for others, provided that the original source and author(s) are credited.Zhonghua Wei, Guodong Ren
