# Supplementary material for: ﻿Two new species of the genus Laena (Coleoptera, Tenebrionidae, Lagriinae) from northern Sichuan in China based on morphological and molecular data
Source: Zookeys. 2023 Aug 1;1173:71–83. doi: 10.3897/zookeys.1173.103125 (PMC10410390; doi:10.3897/zookeys.1173.103125)
Supplement: Supplementary material 1 — Supplementary information [file zookeys-1173-071_article-103125__-s001.zip › supplementary/Table S1.docx]

Table S1. Information of Laenini species for the molecular analyses based on *COI*.

| **Taxon** | **Sample code** | **GenBank**  **Accession** | **Locality** | **References** |
| --- | --- | --- | --- | --- |
| *Laena haigouica* | SCN2 |  | 2022.VII.23, China, Sichuan, Songpan, Huanglong, Dawan Village, elev. 2920 m | Current study |
| *Laena kangdingica* | SCN3 |  | 2022.VIII.5, China, Sichuan, Yajiang, Waduozhen, elev. 2600 m | Current study |
| *Laena bowaica* | SCN4-1 |  | 2022.VIII.1, China, Sichuan, Danba, Bianerxiang, Erwacao Village, elev. 2470 m | Current study |
| *Laena bowaica* | SCN4-1 |  | 2022.VIII.1, China, Sichuan, Danba, Bianerxiang, Erwacao Village, elev. 2470 m | Current study |
| *Laena bifovedata* | SCN5-1 |  | 2022.VIII.26, China, Gansu, Longnan, Taopingxiang Taoping Forestry Farm, elev. 2576 m | Current study |
| *Laena bifovedata* | SCN5-2 |  | 2022.VIII.26, China, Gansu, Longnan, Taopingxiang Taoping Forestry Farm, elev. 2576 m | Current study |
| *Laena puetzi* | SCN6 |  | 2022.VII.31, China, Sichuan, Jinchuan, Dusongxiang, Dusonggou, elev. 2264 m | Current study |
| *Laena maowenica* | SCN7-1 |  | 2022.VII.20, China, Sichuan, 6 KM Eastern Mao County, elev. 1896 m | Current study |
| *Laena maowenica* | SCN7-2 |  | 2022.VII.20, China, Sichuan, 6 KM Eastern Mao County, elev. 1896 m | Current study |
| *Laena fengileana* | SCN8 |  | 2022.VII.22, China, Sichuan, Songpan, Mounigou, Shangzhai Village, elev. 3070 m | Current study |
| *Laena becvari* | SCN12-1 |  | 2022.VIII.7, China, Sichuan, Litang, Junba, elev. 3050 m | Current study |
| *Laena becvari* | SCN12-2 |  | 2022.VIII.7, China, Sichuan, Litang, Junba, elev. 3050 m | Current study |
| *Laena mounigouica* sp. nov. | SCN14-1 |  | 2022.VII.21, Sichuan, Songpan, Mounigou, Tuguanzhai, elev. 2978 m | Current study |
| *Laena mounigouica* sp. nov. | SCN14-2 |  | 2022.VII.21, Sichuan, Songpan, Mounigou, Tuguanzhai, elev. 2978 m | Current study |
| *Laena shaluica* | SCN18-1 |  | 2022.VIII.5, China, Sichuan, Yajiang, Waduozhen, Ridui Village, elev. 3100 m | Current study |
| *Laena shaluica* | SCN18-2 |  | 2022.VIII.5, China, Sichuan, Yajiang, Waduozhen, Ridui Village, elev. 3100 m | Current study |
| *Laena barkamica* | SCN19-1 |  | 2022.VII.26, China, Sichuan, Heishui, Yangyong, Hade Village, elev. 2600 m | Current study |
| *Laena barkamica* | SCN19-2 |  | 2022.VII.26, China, Sichuan, Heishui, Yangyong, Hade, elev. 2600 m | Current study |
| *Laena fengileana* | SCN21-1 |  | 2022.VII.23, China, Sichuan, Songpan, Huanglongxiang, Dawan Village, elev. 2920 m | Current study |
| *Laena fengileana* | SCN21-2 |  | 2022.VII.23, China, Sichuan, Songpan, Huanglongxiang, Dawan Village, elev. 2920 m | Current study |
| *Laena yajiangica* | SCN22-1 |  | 2022.VIII.4, China, Sichuan, Daofu, Xiatuoxiang, Yiwu Village, elev. 2780 m | Current study |
| *Laena yajiangica* | SCN22-2 |  | 2022.VIII.4, China, Sichuan, Daofu, Xiatuoxiang, Yiwu Village, elev. 2780 m | Current study |
| *Laena dentithoraxa* sp. nov. | SCN28 |  | 2022.VIII.6, China, Sichuan, Yajiang Yizhan, elev. 2800 m | Current study |
| *Laena puetzi* | SCN29-1 |  | 2022.VII.29, China, Sichuan, Barkman, Shaerzong, Dazatou Village, elev. 2690 m | Current study |
| *Laena puetzi* | SCN29-2 |  | 2022.VII.29, China, Sichuan, Barkman, Shaerzong, Dazatou Village, elev. 2690 m | Current study |
| *Hypolaenopsis nomurai* | SCN10 |  | 2022.VII.23, China, Sichuan, Songpan, Huanglongxiang, Dawan Village, elev. 2920 m | Current study |
| *Hypolaenopsis* sp. | SCN13 |  | 2022.VII.26, China, Sichuan, Heishui, Yangrong, Hade Village, elev. 2600 m | Current study |
| *Hypolaenopsis hongyuanica* | SCN25 |  | 2022.VII.26, China, Sichuan, Hongyuan, Shuajingsi, elev. 3160 m | Current study |
| *Grabulax darlingtoni* | n/a | KU233834 | Colombia, Sierra Nevada de Santa Marta | [44] |
| *Anaedus brunneus* | n/a | MN448231 | n/a | Unpublished |
